# Vibrational spectroscopy and artificial intelligence for dynamic phenotypic characterization of antimicrobial resistance

**DOI:** 10.3389/fcimb.2026.1927600

**Published:** 2026-10-21

**Authors:** Claudia P. Barrera Patiño, Jennifer M. Soares, Kate C. Blanco, Vanderlei S. Bagnato

**Affiliations:** 1 São Carlos Institute of Physics, University of São Paulo, IFSC – USP, São Carlos, Brazil; 2 Biomedical Engineering, Texas A&M University, College, Station, TX, United States

**Keywords:** antimicrobial resistance, artificial intelligence - AI, dynamic phenotyping, FTIR spectroscopy, machine learning algorithms

## Abstract

The rapid emergence of antimicrobial resistance has created an urgent need for diagnostic technologies capable of rapidly identifying resistant phenotypes and informing precision antimicrobial therapy. Conventional antimicrobial susceptibility testing methods remain clinically reliable; however, they are limited by relatively long turnaround times and restricted physiological resolution. In recent years, vibrational spectroscopy techniques have emerged as promising platforms for rapid phenotypic microbiology because they generate label-free biochemical fingerprints associated with proteins, lipids, nucleic acids, carbohydrates, and membrane biomolecules. When integrated with machine learning and artificial intelligence, these technologies could provide highly sensitive tools for rapid bacterial classification, resistance prediction, and monitoring of dynamic physiological adaptation during antibiotic exposure. This review discusses the integration of chemometrics, machine learning, and deep learning algorithms with spectroscopic datasets, emphasizing both predictive performance and biological interpretability. It is highly useful for bacterial identification, biochemical characterization, and resistance profiling across multiple microbial species. Major controversies regarding genotype-versus-phenotype diagnostics, reproducibility, standardization, and AI explainability are also examined. Finally, we highlight current research gaps and future directions, including single-cell spectroscopy, multimodal phenotyping, explainable AI frameworks, and approaches in systems microbiology. Taken together, the available evidence indicates that integrating vibrational spectroscopy with artificial intelligence could substantially reshape future antimicrobial resistance diagnostics and mechanistic microbiology.

## 1 Introduction

Antimicrobial resistance (AMR) is one of the greatest challenges to modern medicine and global public health, significantly compromising the effectiveness of currently available antibiotics and increasing morbidity, mortality, length of hospital stay, and healthcare costs worldwide (Laxminarayan et al., 2013; Holmes et al., 2016; Murray et al., 2022). The excessive use of antibiotics in clinical, agricultural, and environmental settings has accelerated the emergence and dissemination of multidrug-resistant microorganisms through selective evolutionary pressure (Davies and Davies, 2010; Kandavalli et al., 2022; Murray et al., 2022). In response to the growing global burden of AMR, the World Health Organization (WHO) established international strategic action plans focused on surveillance, prevention, antimicrobial stewardship, and development of innovative diagnostic and therapeutic technologies (Murugaiyan et al., 2022). The major goals of these initiatives are to reduce the emergence and spread of resistance, preserve the efficacy of currently available therapies, and minimize the impact of AMR on human and animal health and global socioeconomic systems.

The AMR crisis has been approached from two complementary perspectives. The first involves the development of rapid and reliable diagnostic technologies capable of detecting resistant phenotypes at early stages of infection (Jorgensen and Ferraro, 2009; van Belkum et al., 2019; Gajic et al., 2022; Reszetnik et al., 2024). The second focuses on the discovery of new antimicrobial compounds, novel therapeutic strategies, and AI drug development platforms (Jiménez-Luna et al., 2020; Schneider et al., 2020; Stokes et al., 2020; Allenspach et al., 2024). Increasing evidence indicates that bacterial resistance cannot be fully explained by isolated genetic determinants, but rather emerges from highly interconnected evolutionary, metabolic, and physiological adaptation processes involving membrane remodeling, stress response activation, metabolic plasticity, and regulatory network reorganization (Blair et al., 2015; Baquero et al., 2021; Smith et al., 2023; Shepherd et al., 2024).

Together, these observations suggest that bacterial responses to antimicrobial pressure should be understood as dynamic processes involving continuous physiological adaptation. In this review, *dynamic antimicrobial response phenotyping* is used as an integrative analytical framework for monitoring time dependent bacterial responses to antimicrobial exposure. It does not imply biological or operational equivalence among resistance, tolerance, persistence, heteroresistance, biofilm-associated survival, and reversible stress responses (El-Halfawy and Valvano, 2015; Brauner et al., 2016; Ciofu et al., 2022).

Conventional microbiological methods remain essential in clinical laboratories; however, their relatively long turnaround times frequently delay targeted antimicrobial therapy and contribute to empirical antibiotic overexposure (van Belkum et al., 2020; Salam et al., 2023). Simultaneously, there is increasing global concern regarding the accelerated emergence of multidrug-resistant organisms, particularly among ESKAPE pathogens (*Enterococcus faecium*, *Staphylococcus aureus*, *Klebsiella pneumoniae*, *Acinetobacter baumannii*, *Pseudomonas aeruginosa*, and *Enterobacter* spp.), which are responsible for a large proportion of hospital-acquired infections worldwide (Alekshun and Levy, 2007; De Oliveira et al., 2020; Miller and Arias, 2024). Recent evidence demonstrates that ESKAPE pathogens exhibit remarkable adaptive capacity and rapidly develop resistance against both clinically available antibiotics and compounds currently under development (Vale de Macedo et al., 2021; Smith et al., 2023; Miller and Arias, 2024; Daruka et al., 2025). In turn, rapid identification of resistant strains has become fundamental for optimizing antimicrobial administration and improving precision infectious disease management (Wenning and Scherer, 2013; Hughes and Andersson, 2017).

These limitations have renewed interest in alternative phenotypic methodologies, among which vibrational spectroscopy has received considerable attention because of its ability to capture the integrated biochemical state of microbial cells (Wenning and Scherer, 2013; Kochan et al., 2019; Ribeiro da Cunha et al., 2020). Techniques such as Fourier transform infrared (FTIR) and Raman spectroscopy enable the acquisition of label-free biochemical fingerprints directly from bacterial cells by analyzing molecular vibrations associated with proteins, nucleic acids, lipids, phospholipids, and carbohydrates (Prada et al., 2022; Rodriguez et al., 2023; Barrera Patiño et al., 2024a, 2025; Bonner et al., 2025). These spectral signatures reflect dynamic biochemical alterations induced by antimicrobial stress and may therefore provide integrated information on bacterial physiology, metabolic adaptation, membrane remodeling, and stress response organization (Arzanlou et al., 2017; Yi et al., 2022; Liu et al., 2024). FTIR spectroscopy appears particularly well suited for monitoring antimicrobial-induced metabolic changes (Kochan et al., 2019; Ribeiro da Cunha et al., 2020; Barrera Patiño et al., 2025; Bonner et al., 2025). This is because specific spectral regions associated with proteins, phospholipids, and nucleic acids often show significant alterations during bacterial adaptation.

The integration of artificial intelligence (AI), chemometrics, and machine learning (ML) algorithms with vibrational spectroscopy has substantially expanded the analytical potential of spectroscopic microbiology (Kaye and Pogue, 2015; Anahtar et al., 2021; Barrera-Patiño et al., 2023; Theodosiou and Read, 2023). ML models, including support vector machines, neural networks, deep learning architectures, and chemometric frameworks, can process high-dimensional spectral datasets and identify subtle nonlinear biochemical patterns associated with resistant phenotypes (Burgula et al., 2007; Kelly et al., 2011; Athamneh et al., 2014; Wang et al., 2018). Recent FTIR studies have demonstrated that machine learning algorithms can discriminate antibiotic-resistant bacteria by analyzing specific biochemical absorption bands and dynamic spectral covariance structures associated with resistance progression (Kochan et al., 2019; Bonner et al., 2025). Importantly, these approaches not only enable rapid identification of resistant phenotypes but also allow investigation of adaptive microbial dynamics, transitional physiological states, and temporal resistance evolution during antimicrobial exposure.

Current observations raise the possibility that spectroscopic covariance structures may encode hidden physiological architectures associated with bacterial adaptation and antimicrobial stress responses (Barrera-Patiño et al., 2023; Barrera-Patiño CP. et al., 2024; Barrera Patiño et al., 2025; Bonner et al., 2025). As a result, vibrational spectroscopy combined with AI may support a new generation of clinical microbiology workflows centered on rapid, nondestructive, mechanistically informative, and data-driven diagnostics (Kaprou et al., 2021; Jeon et al., 2022; Kim et al., 2022; Saikia et al., 2025). These technologies could significantly enhance precision antimicrobial stewardship. More importantly, they may enable real-time monitoring of bacterial adaptation and provide new insights into how resistance evolves.

Recent FTIR studies suggest that covariance patterns among spectral features may reflect coordinated biochemical changes associated with antimicrobial adaptation. As a result, vibrational spectroscopy combined with AI may support a new generation of clinical microbiology workflows centered on rapid, nondestructive, mechanistically informative, and data-driven diagnostics (Kaprou et al., 2021; Jeon et al., 2022; Kim et al., 2022; Saikia et al., 2025). These technologies could significantly enhance precision antimicrobial stewardship. More importantly, they may enable real time monitoring of bacterial adaptation and provide new insights into how resistance evolves.

## 2 Background

### 2.1 Operational definitions of antimicrobial-response phenotypes

Resistance, tolerance, persistence, heteroresistance, adaptive resistance, biofilm-associated tolerance, and antimicrobial stress responses represent distinct biological phenomena and should not be interpreted as interchangeable outcomes (Fernández et al., 2011; Brauner et al., 2016; Balaban et al., 2019; Ciofu et al., 2022). Their characterization requires different experimental endpoints, ranging from minimum inhibitory concentration (MIC) determination and clinical breakpoint classification to time-kill kinetics, population analysis profiling, and biofilm survival assays (International Organization for Standardization, 2021; Zimmer et al., 2024; James et al., 2025). **Table 1** summarizes the operational definitions and principal experimental endpoints adopted in this review.

**Table 1:** Operational definitions of antimicrobial response phenotypes.

Phenomenon	Operational definition	Experimental endpoint
Resistance	Ability to grow at higher antimicrobial concentrations.	MIC and classification according to clinical breakpoints (Zimmer et al., 2024; James et al., 2025).
Tolerance	Prolonged survival during bactericidal exposure without a necessary increase in MIC.	Time-kill curves, MDK, and viable CFU counts (International Organization for Standardization, 2021; Zimmer et al., 2024; James et al., 2025).
Persistence	Transient survival of a phenotypic subpopulation.	Biphasic killing kinetics and regrowth after antimicrobial removal (Balaban et al., 2019).
Heteroresistance	Presence of a subpopulation with reduced susceptibility within an apparently susceptible population.	Population analysis profiling and resistant-subpopulation frequency (El-Halfawy and Valvano, 2015; Band and Weiss, 2019).
Adaptive resistance	Transient and reversible reduction in susceptibility following antimicrobial exposure or an environmental stimulus.	MIC before and after exposure and demonstration of reversion following stimulus removal (Fernández et al., 2011; Fernández and Hancock, 2012).
Biofilm-associated tolerance	Survival depends on biofilm architecture, extracellular matrix, and metabolic heterogeneity.	MBIC, MBEC, viable CFU counts, and viability assays (Ciofu et al., 2022).
Antimicrobial stress response	Antimicrobial-induced physiological change that does not necessarily indicate resistance.	Physiological or spectroscopic biomarkers associated with cellular stress (Jensen et al., 2017).

CFU, colony-forming units; MBEC, minimum biofilm eradication concentration; MBIC, minimum biofilm inhibitory concentration; MDK, minimum duration for killing; MIC, minimum inhibitory concentration.

Recent advances have introduced new techniques to detect and analyze AMR, emphasizing the need to understand underlying mechanisms and reduce identification and AMR diagnostic time (van Belkum et al., 2020; Arnold et al., 2025). One critical example is the need for a rapid and simple method for the accurate identification of Methicillin-resistant S. aureus (MRSA) (Wang et al., 2021; Chen et al., 2022), polymerase chain reaction (PCR), enzyme-linked immunosorbent assay (ELISA) (Chen et al., 2019; Uysal Ciloglu et al., 2020), and DNA sequencing (Wang et al., 2021) remain standard. More recently, matrix-assisted laser desorption/ionization time-of-flight mass spectrometry (MALDI-TOF MS) (Sandrin et al., 2013; Jeon et al., 2022), flow cytometry, and micro-and nano-droplets (Liu et al., 2016) techniques have been developed for rapid antibiotic susceptibility testing (Zhang P. et al., 2022).

Recent advances emphasize alternative and complementary strategies, including antimicrobial peptides, bacteriophage therapy, CRISPR systems for antimicrobials, and nanotechnology drug delivery, which aim to overcome limitations of traditional antibiotics (Beavogui et al., 2024; Khan et al., 2024; Kuchay, 2024). In addition, artificial intelligence and machine learning are increasingly applied to accelerate drug discovery, predict resistance evolution, and enhance diagnostic accuracy (Jiménez-Luna et al., 2020; Stokes et al., 2020; Kim et al., 2022). This has driven interest in integrating optical and spectroscopic tools with machine learning for faster, safer, and cost-effective diagnostics (Wang et al., 2018; Yi et al., 2022). At their core, statistical classification methods in machine learning algorithms enhance pattern recognition in microbial samples, enabling precise differentiation between strains.

One of the most critical barriers is the slow, labor-intensive nature of traditional antimicrobial susceptibility testing (AST), which delays targeted therapy and promotes empirical antibiotic use (Reszetnik et al., 2024; Bilgin et al., 2025). Additionally, the declining antibiotic discovery and the spread of multidrug-resistant organisms further complicate treatment strategies (Miethke et al., 2021; Murray et al., 2022; Maharramov et al., 2025) (Miethke et al., 2021; Murray et al., 2022; Daruka et al., 2025). Antimicrobial resistance should therefore be regarded as a multifactorial and dynamic phenomenon involving genomic, phenotypic, clinical, and environmental determinants.

### 2.2 Comparative capabilities of vibrational-spectroscopy platforms

Vibrational spectroscopy platforms provide complementary rather than interchangeable information on microbial composition and antimicrobial responses. Because infrared absorption and Raman scattering follow different molecular selection rules and respond differently to water, fluorescence, substrates, and sample thickness, each platform examines bacterial physiology at a different scale, from population level biochemical fingerprints to single-cell metabolism and nanoscale molecular organization (Baker et al., 2014; Baker et al., 2018; Morais et al., 2019).

ATR-FTIR enables rapid biochemical fingerprinting with minimal sample preparation, although spectral quality depends on biomass, hydration, substrate properties, and sample-to-crystal contact. Transmission FTIR requires thin and homogeneous samples, whereas FTIR micro spectroscopy provides spatial information from microbial aggregates or microcolonies but remains limited by mid-infrared spatial resolution (Baker et al., 2014; Baker et al., 2018; Morais et al., 2019; Kochan et al., 2020). Conventional Raman spectroscopy is less affected by water and can analyze individual cells, but weak signals, fluorescence, photodamage, and longer acquisition times remain important limitations. SERS improves sensitivity but depends strongly on substrate reproducibility and nanoparticle organization (Baker et al., 2018; Uysal Ciloglu et al., 2020). Resonance Raman provides greater molecular selectivity for specific chromophores but does not capture the complete cellular biochemical fingerprint (Kunapareddy et al., 2015).

Single-cell Raman approaches, including Deuterium isotope probing and stimulated Raman scattering, can detect antimicrobial-induced metabolic changes before visible population growth and may help investigate bacterial heterogeneity and rare subpopulations. However, their results require validation against MIC, time-kill assays, and accepted susceptibility categories (Tao et al., 2017; Zhang et al., 2020). AFM-IR provides nanoscale chemical information from individual bacteria, but its low throughput and instrumental complexity currently limit its use mainly to mechanistic studies (Mayet et al., 2010; Kochan et al., 2018).

Time-resolved FTIR studies demonstrate that antimicrobial responses can be monitored spectroscopically over time. Nevertheless, their results cannot be automatically generalized to Raman, SERS, resonance Raman, single-cell Raman, or AFM-IR. Platform of specific analytical and biological validation is therefore required before spectral signatures can be interpreted as indicators of antimicrobial susceptibility or applied to direct clinical specimens (Baker et al., 2016; Baker et al., 2018; Uysal Ciloglu et al., 2020; Zhou et al., 2020). **Table 2** summarizes the main analytical characteristics, practical limitations, and potential clinical applications of the principal vibrational spectroscopy platforms used for microbial phenotyping.

**Table 2:** Comparative analytical characteristics and translational potential of vibrational spectroscopy platforms used for microbial phenotyping.

Platform	Spatial resolution and analytical scale	Main interferences and substrate effects	Sample preparation and culture requirements	Acquisition time and throughput	Reproducibility and analytical complexity	Suitability for direct clinical specimens
ATR-FTIR (Baker et al., 2014; Morais et al., 2019; Chen et al., 2022)	Mainly population-level biochemical fingerprinting and micrometer-scale sampling depth.	Water interference remains relevant. Results depend on sample and crystal contact, biomass, hydration, pressure, and crystal cleanliness.	Concentrated biomass is usually deposited onto the ATR crystal, and prior drying and culture or enrichment are commonly required.	Seconds to minutes per spectrum, relatively high throughput.	Moderate-to-high reproducibility with standardized preparation and quality control; relatively low complexity.	Good with standardized protocols, moderate complexity, direct use limited by matrix interference and low biomass.
Transmission FTIR (Baker et al., 2014; Baker et al., 2018; Morais et al., 2019)	Population-level fingerprinting: no intrinsic single-cell resolution without microscopy.	Strong water interference; sensitive to optical path length, film thickness, scattering, and IR window properties.	Requires thin, homogeneous samples, frequently prepared as dried films on IR-transparent substrates.	Seconds to minutes per spectrum, potentially high throughput.	Good reproducibility is possible, although sample thickness and hydration can introduce substantial variability.	Limited for untreated specimens; more suitable after isolation, concentration, or culture.
FTIR microspectroscopy (Baker et al., 2014; Kochan et al., 2020; Jaber et al., 2026)	Spatially resolved analysis of microbial aggregates and microcolonies, diffraction-limited micrometer-scale resolution.	Affected by water, scattering, sample thickness, focus, and substrate composition.	Deposition on IR-compatible substrates, drying or controlled hydration, and microbial enrichment are generally required.	Point spectra may be rapid, but spectral mapping can take minutes to hours and has low to moderate throughput.	Greater instrumental and preprocessing complexity than bulk FTIR, sensitive to focus and spatial heterogeneity.	Emerging potential after isolation or enrichment remains difficult to analyze directly in complex matrices.
Conventional Raman spectroscopy (Tao et al., 2017; Baker et al., 2018; Zhang et al., 2020)	Optical resolution is commonly sufficient for individual bacterial cells and single-cell biochemical fingerprinting.	Low water interference, but fluorescence, laser heating, photodamage, and substrate signals can be substantial.	Compatible with wet or dried cells, microbial isolation or concentration is generally necessary.	Seconds to minutes per cell, low to moderate throughput without automation.	Moderate reproducibility requires calibration, fluorescence correction, and control of laser and acquisition parameters.	Promising for concentrated or minimally processed specimens, but low bacterial load and fluorescence remain barriers.
SERS (Baker et al., 2018; Uysal Ciloglu et al., 2020)	Optical resolution with signal enhancement localized at nanoscale electromagnetic hotspots high sensitivity.	Strongly dependent on metallic substrate composition, nanoparticle aggregation, hotspot distribution, and analyte surface interactions.	Requires a reproducible SERS-active substrate; washing, isolation, or concentration may be necessary.	Seconds per spectrum; potentially medium-to-high throughput after substrate preparation.	High sensitivity, but inter-substrate and inter-batch variability remain major challenges.	Promising for low concentration detection, but substrate standardization and clinical matrix effects require validation.
Resonance Raman spectroscopy (Kunapareddy et al., 2015; Baker et al., 2018)	Selective enhancement of vibrations from specific chromophores; cell-scale optical resolution.	Water interference is low, but fluorescence and photodamage depend on excitation wavelength and chromophore properties.	Limited preparation may be required, but the biological target must contain an appropriate chromophore; culture or enrichment is usually necessary.	Seconds to minutes per spectrum; low-to-moderate throughput.	High molecular selectivity, but strong dependence on excitation wavelength, laser stability, and chromophore composition.	Currently, it is more appropriate for targeted mechanistic studies than for broad routine susceptibility testing.
Single-cell Raman and Raman-DIP (Tao et al., 2017; Zhang et al., 2020)	Individual-cell metabolic and biochemical phenotyping resolves cellular heterogeneity and rare subpopulations.	Affected by fluorescence, cell-to-cell variability, laser exposure, and substrate or matrix background.	Requires isolation or optical localization of individual cells; Raman DIP additionally requires D_2_O exposure; short enrichment may still be necessary.	Spontaneous Raman generally requires seconds to minutes per cell; stimulated Raman is faster, but complete AST workflows require antimicrobial incubation.	High instrumental and analytical complexity; standardized metabolic thresholds and reference AST validation are required.	High translational potential, but direct specimen workflows remain under clinical validation.
AFM-IR (Mayet et al., 2010; Kochan et al., 2018)	Nanoscale chemical mapping of individual bacteria and subcellular regions, with resolution below the optical diffraction limit.	Sensitive to surface roughness, sample thickness, thermal and mechanical properties, tip condition, drift, and substrate selection.	Requires surface immobilization and careful control of sample geometry; specialized configurations are required for hydrated or living cells.	Minutes per spectrum and potentially hours for detailed maps, low throughput.	High instrumental and interpretive complexity, tip and sample interactions require rigorous control.	Currently unsuitable for routine direct clinical analysis, primarily useful for mechanistic and nanoscale validation studies.

Spatial resolution and acquisition time are instrument-and protocol-dependent and should not be interpreted as fixed performance specifications. Direct clinical specimens are samples analyzed with minimal or no culture or enrichment. Abbreviations: AFM-IR, atomic force microscopy infrared spectroscopy; AST, antimicrobial susceptibility testing; ATR-FTIR, attenuated total reflectance Fourier-transform infrared spectroscopy; Raman-DIP, Raman Deuterium Isotope Probing; SERS, Surface-Enhanced Raman Spectroscopy.

### 2.3 Antibiotic targets and associated spectroscopic alterations

The main classes of antibiotics, their primary bacterial targets, and the key biochemical adaptations associated with antimicrobial resistance (AMR) that can be detected using vibrational spectroscopy approaches, such as Fourier transform infrared (FTIR) and Raman spectroscopy (Wenning and Scherer, 2013; Germond et al., 2018; Kochan et al., 2019; Barrera-Patiño et al., 2023; Barrera Patiño et al., 2024a). **Table 3** shows how the physiological remodeling associated with resistance affects multiple biochemical compartments simultaneously, including carbohydrates, lipids, nucleic acids, phospholipids, and proteins, through molecular mechanisms of antibiotic resistance (Germond et al., 2018; Kochan et al., 2019; Barrera Patiño et al., 2024a, 2025). β-lactams and glycopeptides predominantly induce changes in cell wall architecture and peptidoglycan organization (Dörries et al., 2014; Magréault et al., 2022), while polymyxins and lipopeptides (Sandrin et al., 2013) strongly affect membrane integrity (De Carvalho and Caramujo, 2018; Gauba and Rahman, 2023; Sidders et al., 2023) and phospholipid composition (Mishra et al., 2011; Kochan et al., 2019). Antibiotics that target nucleic acid synthesis, such as fluoroquinolones and rifamycins, frequently induce spectral signatures associated with DNA damage, transcriptional stress, and regulatory adaptation (Athamneh et al., 2014; Dawan and Ahn, 2022). In contrast, protein synthesis inhibitors, including aminoglycosides, tetracyclines, and macrolides, are associated with alterations in ribosomal proteins, stress response pathways, and metabolic compensation mechanisms (Allison et al., 2011; Hughes and Andersson, 2017). Relevant information on antibiotic classes, biochemical targets related to antimicrobial resistance, and the main adaptive effect detectable by vibrational spectroscopy is summarized in **Table 3**.

**Table 3:** Classes of antibiotics, biochemical targets related to antimicrobial resistance (AMR), and the relevance of vibrational spectroscopy.

Antibiotic class	Main bacterial target	Main biochemical changes related to antimicrobial resistance	Main adaptive effect detected by FTIR/Raman spectroscopy
β-lactams (Bush and Bradford, 2020, Sauvage et al., 2008, Drawz and Bonomo, 2010)	Peptidoglycan synthesis, cell wall synthesis, and PBPs.	Cell wall remodeling, altered peptidoglycan architecture, and β-lactamase activity.	Strong changes in carbohydrate and phosphate regions, protein bands linked to cell wall stress.
Glycopeptides (Arthur and Courvalin, 1993; Binda et al., 2014)	Cell wall terminal motifs and peptidoglycan terminal motifs.	Altered cell wall precursors.	Carbohydrate and cell wall spectral signatures changes.
Aminoglycosides (Peyrusson et al., 2020, Krause et al., 2016, Becker and Cooper, 2013)	Ribosomal protein synthesis.	Oxidative stress, membrane permeability, and altered protein synthesis.	Protein, metabolic, and membrane spectral signatures change.
Macrolides (Vázquez-Laslop and Mankin, 2018a, 2018b, Champney and Burdine, 1995)	50S ribosomal subunit.	Ribosomal methylation and efflux activation.	Protein vibrational signatures.
Tetracyclines (Chopra and Roberts, 2001, Brodersen et al., 2000, Wilson, 2014, Farrar et al., 2025)	30S ribosomal subunit.	Efflux pumps and ribosomal protection proteins.	Protein and membrane spectral variation.
Fluoroquinolones (Kochan et al., 2019, Athamneh et al., 2014, Germond et al., 2018, Drlica and Zhao, 1997)	DNA gyrase and topoisomerase IV.	DNA damage response, transcriptional stress and adaptive regulation.	Nucleic acid spectral shifts and stress response signatures.
Polymyxins (Velkov et al., 2010, Olaitan et al., 2014, Trimble et al., 2016)	Outer membrane and lipid A.	Membrane charge modification and phospholipid remodeling.	Strong lipid and phospholipid spectral changes.
Lipopeptides (Kochan et al., 2019, Mishra et al., 2011, Gaupp et al., 2012)	Cytoplasmic membrane.	Membrane depolarization and lipid redistribution.	Membrane spectral signatures, lipid and phospholipid spectral alterations.
Rifamycins (Campbell et al., 2001, Floss and Yu, 2005)	RNA polymerase.	Transcriptional adaptation and metabolic compensation.	Protein and nucleic acid spectral changes linked to transcriptional stress.
Sulfonamides and Trimethoprim (Sköld, 2000, Huovinen et al., 1995)	Folate metabolism.	Altered nucleotide synthesis and metabolic pathways.	Broad metabolic fingerprint changes.
Biofilm AMR (Peyrusson et al., 2020, Flemming et al., 2023, Anderson and O’Toole, 2008)	Multicellular adaptive state and matrix-protected tolerance.	Extracellular polysaccharides, matrix proteins, and dormant physiology.	Carbohydrate, lipid, and protein persistence and tolerance signatures.
Multidrug resistance (MDR) (Holmes et al., 2016, Baquero et al., 2021, De Oliveira et al., 2020, Alekshun and Levy, 2007, Magiorakos et al., 2012)	Multiple cellular targets.	Efflux activation, membrane remodeling, and stress response integration.	Integrated spectral covariance changes across proteins, lipids, carbohydrates, and nucleic acids.

It is important to emphasize that AMR would not be interpreted exclusively as a genetic phenomenon, but rather as an integrated systemic adaptive process involving coordinated biochemical remodeling in multiple cellular structures and metabolic pathways (Motta et al., 2015; Sandoval‐Motta and Aldana, 2016; Smith et al., 2023). Several recent FTIR studies have demonstrated that resistant phenotypes can be discriminated using dynamic spectral covariance structures that reflect membrane remodeling, efflux activation, metabolic plasticity, persistence in biofilms, and regulatory network reorganization (Peyrusson et al., 2020; Soares et al., 2022; Li et al., 2024). In addition, temporal spectroscopic analyses can reveal progressive trajectories of resistance evolution, allowing the investigation of transitional physiological states and adaptive microbial dynamics over time (Andersson et al., 2020; Shepherd et al., 2024; Barrera Patiño et al., 2025; Bonner et al., 2025). Taken together, the available evidence suggests that spectroscopic microbiology progressively moves from a promising experimental approach toward a clinically relevant translational platform.

## 3 Discussion

The rapid global expansion of AMR has intensified the need for diagnostic methodologies capable of detecting bacterial adaptation beyond conventional culture-dependent approaches. Thus, modern microbiology has progressively shifted toward multidimensional analytical frameworks integrating vibrational spectroscopy, chemometrics, artificial intelligence (AI), and systems biology approaches (MacLean et al., 2010; Barrera Patiño et al., 2025; Bonner et al., 2025). An increasingly accepted view is that resistance and tolerance emerge from coordinated physiological adaptation involving metabolic rewiring, stress responses, and membrane remodeling rather than from isolated genetic determinants alone (Corona and Martinez, 2013; Ribeiro da Cunha et al., 2020) (Corona and Martinez, 2013; Ribeiro da Cunha et al., 2020). Recent investigations demonstrated that FTIR spectroscopy combined with machine learning algorithms could discriminate resistant bacterial phenotypes; it suggested that the spectral signatures associated with resistance may reflect metabolic adaptation processes rather than merely the presence of canonical resistance genes (Sulaiman and Lam, 2021; Barrera-Patiño et al., 2023; Barrera Patiño et al., 2024b; Bonner et al., 2025). More recently, time-resolved FTIR analyses combined with principal component analysis (PCA) demonstrated that bacterial resistance progression may be dynamically monitored through spectral covariance trajectories during antibiotic exposure (Jolliffe, 2002; Barrera Patiño et al., 2025; Bonner et al., 2025). These observations support the hypothesis that AMR is a continuous adaptive physiological process rather than a static binary state.

In contrast, another important research perspective argues that genotypic methodologies remain more reproducible, scalable, and clinically actionable than phenotypic spectroscopic approaches (Boolchandani et al., 2019; Jeon et al., 2022). Whole genome sequencing, PCR resistance detection, and bioinformatic indicators provide highly specific identification of known resistance determinants and epidemiological markers (Gussow et al., 2020; Tang et al., 2022). Although genotype-based methods provide valuable information on resistance determinants, accumulating evidence suggests they often fail to capture the physiological complexity that ultimately determines antimicrobial susceptibility. This is partly because bacterial susceptibility is strongly influenced by environmental conditions, metabolic state, membrane energetics, and cellular heterogeneity (Carvalho et al., 2018; Batchelder et al., 2023; Cotten and Davis, 2023). This controversy remains central in contemporary AMR diagnostics: while genomic approaches identify resistance potential, phenotypic methodologies reveal the real-time physiological state of microorganisms under antimicrobial stress (Boolchandani et al., 2019; Morais et al., 2019; van Belkum et al., 2020).

Recent advances in AI and deep learning have attempted to bridge these paradigms by integrating complex phenotypic datasets with predictive computational models (Ching et al., 2018; Anahtar et al., 2021; Theodosiou and Read, 2023; Zagajewski et al., 2023). Neural networks, convolutional neural networks (CNNs), support vector machines (SVMs), random forests, and chemometric algorithms are increasingly applied to microbiological datasets because of their ability to process high-dimensional nonlinear spectral structures (Salcedo‐Sanz et al., 2014; Theodosiou and Read, 2023; Brizuela et al., 2025; Przymus et al., 2025). In particular, deep learning-assisted single-cell phenotyping demonstrated that image analysis with CNN can identify antibiotic susceptibility phenotypes within minutes by detecting subtle subcellular morphological alterations associated with antimicrobial stress (Baltekin et al., 2017; Lugagne et al., 2020; Wu and Gadsden, 2023). These findings raise the intriguing possibility that bacterial adaptation may become detectable before overt growth inhibition occurs (Andersson, 2003; Allison et al., 2011; Levin-Reisman et al., 2017; Radlinski et al., 2019).

### 3.1 From spectral patterns to biological insight

Computational methods used with vibrational spectroscopy are complementary rather than interchangeable. Their suitability depends on the biological question, dataset size and structure, expected nonlinearity, and required level of interpretation. PCA and HCA are valuable for exploratory analysis, visualization of spectral variation, and detection of clusters or temporal trajectories. However, because they are unsupervised, apparent group separation should not be interpreted as predictive classification without independent supervised validation (Ringnér, 2008; Brereton and Lloyd, 2014; Bro and Smilde, 2014).

Linear supervised methods such as LDA and PLS-DA can provide relatively transparent models and are useful when the number of independent biological samples is limited and class separation is approximately linear. Loadings and regression coefficients may help identify influential spectral regions, although they do not establish that these regions are mechanistic biomarkers. These methods can also overfit when the number of variables greatly exceeds the number of independent samples, particularly when component selection or preprocessing is performed before data partitioning (Brereton and Lloyd, 2014; Morais et al., 2020).

SVM and random forest provide greater flexibility for nonlinear classification. SVM can perform well with high-dimensional spectra and moderately sized datasets, but its performance depends strongly on kernel selection, regularization, and hyperparameter tuning. Random forest accommodates nonlinear relationships and interactions and provides measures of variable importance, but highly correlated spectral bands may distribute importance across several related variables, complicating biochemical interpretation (Cortes and Vapnik, 1995; Breiman, 2001). Both approaches are suitable for bacterial identification and susceptibility classification, although their transferability may decrease when organisms, culture conditions, sample substrates, or instruments differ from those represented during training.

Artificial neural networks and CNNs can learn complex spectral patterns with less dependence on manually selected features. CNNs are particularly suited to recognizing local peak shapes and relationships between adjacent wavenumbers. Nevertheless, these models generally require larger and more diverse datasets, greater computational resources, and rigorous external validation. When applied to small or homogeneous datasets, their flexibility increases the risk of learning strain-specific, laboratory-specific, or instrument-specific signals rather than generalizable resistance phenotypes (Acquarelli et al., 2017; Vabalas et al., 2019). Transfer learning and calibration-transfer strategies may reduce the amount of new data required when models are moved between instruments or experimental settings, but they do not eliminate the need for independent validation (Mishra and Passos, 2021a; Mishra and Passos, 2021b).

Temporal response analysis requires a further distinction between cross-sectional classification and longitudinal modeling. PCA trajectories can visualize coordinated biochemical changes during antimicrobial exposure, whereas supervised time-aware models can predict progression or identify transitional states. These analyses require repeated measurements from independent biological cultures, strains, or isolates at defined time points. Multiple spectra obtained from the same biological sample are technical replicates and should not be split independently between the training and test sets, as this introduces information leakage and artificially inflates performance. Data partitioning should therefore occur at the highest independent biological level, with preprocessing, feature selection, and hyperparameter optimization performed within the training or nested cross-validation loop (Morais et al., 2019; Vabalas et al., 2019).

Explainable AI methods can support biological interpretation by identifying the spectral variables that contribute to model predictions. PCA and PLS loadings, random-forest importance, permutation-based measures, and SHAP values provide different forms of global or sample-level attribution. However, these outputs describe the model’s statistical behavior rather than demonstrating biological causality. SHAP importance, for example, does not by itself prove that a selected wavenumber represents a resistance mechanism, particularly when nearby bands are strongly correlated. Mechanistic interpretation therefore requires confirmation through spectral-band assignment, reference microbiology, functional assays, and complementary molecular or omics measurements (Rudin, 2019; Lundberg et al., 2020).

Accordingly, no single computational approach is optimal for every spectroscopic AMR application. Exploratory chemometrics is appropriate for hypothesis generation and temporal visualization; LDA, PLS-DA, SVM, and random forest are generally suitable for classification with small-to-moderate datasets; deep learning is most justified when large and heterogeneous datasets are available; and explainable AI methods are best used for generating mechanistic hypotheses rather than causal conclusions (Topol, 2019; Rajpurkar et al., 2022). For clinically oriented applications, simpler and intrinsically interpretable models should be preferred when they achieve performance comparable to more complex black-box alternatives (Rudin, 2019). **Table 4** summarizes the principal requirements, strengths, limitations, and most appropriate applications of these computational approaches.

**Table 4:** Critical comparison of computational approaches used with vibrational spectroscopy for microbial phenotyping and antimicrobial-resistance analysis.

Approach	Dataset requirements and robustness	Interpretability	Overfitting risk and computational burden	Transferability	Most appropriate objectives
PCA/HCA (Bro and Smilde, 2014; Morais et al., 2020)	Small to moderate datasets are sensitive to scaling, preprocessing, outliers, and batch effects.	High for scores, loadings, clusters, and broad spectral patterns.	Low-to-moderate risk, low burden, not intrinsically predictive.	Limited when preprocessing, organisms, or instruments differ.	Exploratory analysis, quality control, cluster detection, and temporal trajectories.
LDA/PLS-DA (Brereton and Lloyd, 2014; Morais et al., 2020)	Small to moderate datasets; most robust when dimensionality is reduced, and separation is approximately linear.	Relatively high; coefficients and loadings identify influential variables.	Moderate risk with high variable-to-sample ratios or improper component selection, low burden.	Moderate with standardized acquisition and preprocessing.	Identification, susceptibility classification, and interpretable spectral discrimination.
SVM (Cortes and Vapnik, 1995; Morais et al., 2020)	Effective for high-dimensional, small-to-moderate datasets; robust with appropriate regularization.	Low to moderate, depending on the kernel and supporting interpretation methods.	Moderate-to-high risk during feature and hyperparameter selection; moderate burden.	Moderate; sensitive to domain and instrument shifts.	Binary or multiclass identification and susceptibility classification.
Random forest (Breiman, 2001; Lundberg et al., 2020)	Moderate datasets accommodate nonlinear effects, interactions, and noisy variables.	Moderate through feature importance, although correlated bands complicate interpretation.	Moderate risk, moderate burden.	Moderate, may decline with batch, organism, or instrument shifts.	Classification, nonlinear pattern detection, and preliminary feature ranking.
ANN/CNN (Acquarelli et al., 2017; Mishra and Passos, 2021a)	Preferably large, diverse, and balanced datasets. CNNs learn local spectral features.	Without XAI, learned representations may be difficult to assign biochemically.	High risk in small datasets, high computational and tuning burden.	Potentially improved by transfer learning, but external validation remains essential.	Large-scale classification, automated feature learning, and complex nonlinear patterns.
Temporal or longitudinal models (Vabalas et al., 2019; Barrera Patiño et al., 2025; Bonner et al., 2025)	Repeated measurements from independent biological units and defined time points.	Variable trajectory models are more interpretable than deep sequence models.	Moderate to high risk when within-sample dependence is ignored, moderate to high burden	Currently limited, requires validation across organisms, drugs, protocols, and instruments.	Adaptation trajectories, transitional states, tolerance, persistence, and resistance progression.
XAI and feature attribution methods (Rudin, 2019; Lundberg et al., 2020)	Applied after model development, reliability depends on the underlying model and reference distribution.	High apparent interpretability, but attribution is not equivalent to causality.	Does not independently prevent overfitting, low to moderate additional burden.	Inherits the predictive model’s transfer limitations.	Global- and sample-level explanation, spectral-region prioritization, and mechanistic hypothesis generation.

Dataset size refers to the number of independent biological samples, isolates, strains, or cultures, rather than the total number of technical spectra. Model selection, preprocessing, and feature selection should be nested within the validation procedure. Abbreviations: ANN, artificial neural network; CNN, convolutional neural network; HCA, hierarchical cluster analysis; LDA, linear discriminant analysis; PCA, principal component analysis; PLS-DA, partial least-squares discriminant analysis; SHAP, Shapley additive explanations; SVM, support vector machine; XAI, explainable artificial intelligence.

Explainable artificial intelligence (XAI) provides an important framework for moving beyond predictive accuracy toward biologically interpretable spectroscopic models (Contreras and Bocklitz, 2025). Approaches such as SHapley Additive exPlanations (SHAP), local interpretable model-agnostic explanations (LIME), permutation importance, feature attribution methods, and class activation mapping can help determine which spectral regions contribute most strongly to model predictions (Selvaraju et al., 2017; Contreras and Bocklitz, 2025). In FTIR and Raman applications, these methods may indicate whether classification is primarily influenced by spectral domains associated with proteins, lipids, nucleic acids, carbohydrates, or cell envelope components (Baker et al., 2014; Kuhar et al., 2018; Bellantuono et al., 2023). Nevertheless, XAI outputs should not automatically be interpreted as evidence of causality or as direct identification of molecular mechanisms (Ghassemi et al., 2021). Because individual vibrational bands frequently contain overlapping contributions from several biomolecules, the biological meaning of influential spectral features must be confirmed using complementary biochemical, molecular, or functional experiments (Baker et al., 2014; Kuhar et al., 2018). Thus, XAI is most valuable when used as a hypothesis-generating and model-auditing strategy rather than as a substitute for mechanistic validation (Ghassemi et al., 2021; Contreras and Bocklitz, 2025).

We anticipate that the next phase of development will increasingly focus on combining FTIR spectroscopy with high-throughput and AI-assisted analytical platforms capable of supporting clinical decision-making (Reszetnik et al., 2024; Bilgin et al., 2025; Cesaro et al., 2025). Portable ATR-FTIR systems, hyperspectral imaging, single-cell spectroscopy, and multimodal vibrational platforms integrating FTIR and Raman spectroscopy may substantially improve diagnostic speed and mechanistic resolution (Baker et al., 2018; Kochan et al., 2019; Saikia et al., 2025). Moreover, time-resolved FTIR analyses may enable continuous monitoring of bacterial adaptation trajectories during antimicrobial exposure, facilitating early identification of tolerance, persistence, and resistance progression before classical phenotypic resistance becomes clinically detectable.

Reproducibility also depends on factors that are independent of the selected algorithm. Culture conditions, hydration, incubation time, sample preparation, substrate composition, and instrument settings can introduce spectral variation between experiments and laboratories (Baker et al., 2014; Butler et al., 2016; Novais et al., 2019). Most available studies also include relatively small datasets and limited strain diversity, restricting external validation across organisms, clinical settings, and analytical platforms (Ho et al., 2019; Thomsen et al., 2022). The limited availability of standardized, open-access spectral databases further constrains multicenter comparisons and the development of transferable models (Wang et al., 2021; Kassem et al., 2023).

Another promising direction involves integrating spectroscopy with ecological and evolutionary modeling to investigate how bacterial populations dynamically reorganize under antimicrobial pressure (Hughes and Andersson, 2017; Shepherd et al., 2024; Maharramov et al., 2025). Recent systems-level studies increasingly suggest that bacterial adaptation emerges from highly interconnected biochemical networks involving membrane physiology, transcriptional regulation, oxidative stress responses, and metabolic plasticity (Davies and Davies, 2010; Gaupp et al., 2012; Jensen et al., 2017). In this context, we consider that vibrational spectroscopy is evolving beyond a purely diagnostic tool toward a systems microbiology platform capable of interrogating bacterial adaptation in real time. ​Thus, vibrational spectroscopy is expected to become an essential tool for investigating emerging physiological states under antimicrobial stress. And their relationship with biochemical signatures associated with the MIC across different spectral regions (Barrera Patiño et al., 2025; Bonner et al., 2025).

Future developments are expected to rely on integrated, multidisciplinary approaches combining novel therapeutics, precision diagnostics, antimicrobial stewardship, and global policy coordination. Strategies such as combination therapies, antivirulence approaches, and microbiome interventions will play a central role in mitigating resistance (van Belkum et al., 2020; Miethke et al., 2021; Shepherd et al., 2024). Overall, overcoming AMR will require not a single solution but a convergence of technological, biological, and public health innovations.

### 3.2 Evidence required for translation from spectral promise to clinical practice

Vibrational spectroscopy can detect biochemical changes associated with antimicrobial exposure and resistant phenotypes. However, demonstrating spectral differences under controlled laboratory conditions is not equivalent to providing a clinically actionable antimicrobial susceptibility testing (AST) result. Mechanistic investigations and proof-of-concept classifiers are important developmental stages, but neither alone establishes readiness for therapeutic decision-making (van Belkum et al., 2019; International Organization for Standardization, 2021; Zimmer et al., 2024). Clinical translation first requires comparison with accepted reference AST methods, such as broth microdilution or agar dilution. Spectroscopic predictions should be linked to reference MIC values and interpreted using current clinical breakpoints.

In addition to classification accuracy, studies should report essential and categorical agreement, major and very major errors, confidence intervals, and indeterminate or failed results (International Organization for Standardization, 2021; Zimmer et al., 2024; James et al., 2025). Models that only distinguish between previously labeled susceptible and resistant isolates should therefore be described as susceptibility classifiers rather than as validated AST platforms. Validation should include independent clinical isolates representing diverse species, genetic backgrounds, resistance mechanisms, antimicrobial classes, and MIC distributions, particularly isolates near clinical breakpoints. **Table 5** summarizes the evidence expected at each translational stage. Training and validation data must be separated at the isolate level rather than at the level of technical spectra. External evaluation across institutions, operators, geographic regions, and instruments is also essential to exclude laboratory/or strain specific learning and demonstrate model generalizability (van Belkum et al., 2019; Zimmer et al., 2024).

**Table 5:** Translational stages and evidence required for the clinical implementation of vibrational-spectroscopy-based antimicrobial susceptibility testing.

Translational stage	Primary objective	Minimum evidence required	Appropriate interpretation
1. Mechanistic laboratory investigation (Baker et al., 2014; Butler et al., 2016)	Determine whether antimicrobial exposure, resistance mechanisms, or adaptive states produce measurable spectral changes.	Controlled laboratory strains; defined antimicrobial exposure; temporal or dose-response experiments; spectral assignment; replication; and complementary biochemical, molecular, or functional validation.	The method detects biochemical or physiological responses associated with antimicrobial exposure or resistance. It should not yet be described as an AST platform.
2. Proof of concept susceptibility classification (van Belkum et al., 2019; Yang et al., 2019; Roberts et al., 2021)	Determine whether spectral models can distinguish selected susceptible and resistant isolates.	Reference-labeled isolates; appropriate preprocessing; training and testing separated at the isolate level; internal validation; confidence intervals; and reporting of failed or indeterminate classifications.	The model demonstrates preliminary susceptibility classification under controlled conditions, but clinical generalizability has not been established.
3. Analytical validation (van Belkum et al., 2019; International Organization for Standardization, 2021; Zimmer et al., 2024; James et al., 2025)	Establish whether the method provides accurate, reproducible, and quality-controlled susceptibility results.	Comparison with broth microdilution, agar dilution, or another accepted reference AST method; agreement with MIC values and clinical breakpoints; essential and categorical agreement; major and very major error rates; reproducibility; limits of detection; calibration; spectral-quality criteria; instrument-drift monitoring; locked preprocessing; and model-version control.	The platform has demonstrated analytical performance for a defined antimicrobial combination in the target organism and intended specimen type.
4. Clinical validation (Liu et al., 2016; Yang et al., 2019; Jayakumar et al., 2022)	Determine performance in the intended clinical population, specimen type, and laboratory setting.	Independent clinical isolates representing diverse genetic backgrounds, resistance mechanisms, species, antimicrobial classes, and MIC distributions; isolates close to clinical breakpoints; clinically relevant matrices; low bacterial loads; host cell and matrix interference; polymicrobial specimens; prospective consecutive enrollment; and external multicenter validation across operators, instruments, and institutions.	The method has demonstrated diagnostic performance in its intended use population and setting, but implementation and clinical utility must still be established.
5. Routine clinical implementation (Butler et al., 2016; van Belkum et al., 2019; Sounderajah et al., 2025)	Demonstrate that the technology can be integrated safely and sustainably into routine microbiology workflows and improve patient management.	End-to-end turnaround time measured from specimen receipt; hands-on time; sample-preparation requirements; failure rate; biosafety; cost; reagent and maintenance requirements; laboratory information system integration; antimicrobial stewardship integration; operator training; regulatory compliance; sustainable quality control; prospective clinical utility studies; and assessment of time to effective or optimized therapy.	Technology is approaching or has achieved readiness for routine clinical implementation within its validated intended use.

AST, antimicrobial susceptibility testing; MIC, minimum inhibitory concentration; CLSI, Clinical and Laboratory Standards Institute; ISO, International Organization for Standardization.

Clinically relevant matrices introduce additional challenges. Blood, urine, respiratory secretions, and wound samples may contain host cells, proteins, salts, residual antimicrobials, and other components that interfere with FTIR or Raman measurements (Baker et al., 2016). Low bacterial loads may also require enrichment, concentration, filtration, washing, or short culture steps (Baker et al., 2014; Pahlow et al., 2015). Polymicrobial specimens are particularly challenging because population-level spectra may combine organisms with different susceptibility profiles. A clinically useful method must resolve species-specific responses, operate at the single-cell level, or identify mixed samples as unsuitable for interpretation (Zhang W. et al., 2022).

Analytical validation also requires standardized sample preparation, instrument calibration, spectral quality criteria, controls, and monitoring of instrument drift. FTIR measurements require control of hydration, sample thickness, optical contact, and substrate composition, whereas Raman and SERS workflows must additionally control laser power, fluorescence, photodamage, and substrate or hotspot variability. Preprocessing pipelines and model versions should be locked, with predefined acceptance criteria and routine quality control procedures (Butler et al., 2019; International Organization for Standardization, 2021). Clinical utility also depends on hands-on time, failure rates, biosafety, cost, reagent requirements, and integration with laboratory information systems and antimicrobial stewardship workflows (van Belkum et al., 2019; International Organization for Standardization, 2021; Zimmer et al., 2024). Turnaround time should cover the complete process from specimen receipt to an interpretable result, including culture, isolation, preparation, antimicrobial incubation, and spectral analysis.

Clinical utility also depends on hands-on time, failure rates, biosafety, cost, reagent requirements, and integration with laboratory information systems and antimicrobial-stewardship workflows (van Belkum et al., 2019; International Organization for Standardization, 2021; Zimmer et al., 2024). Turnaround time should cover the complete process from specimen receipt to an interpretable result, including culture, isolation, preparation, antimicrobial incubation, and spectral analysis. Accordingly, most current vibrational spectroscopy studies should be considered mechanistic or proof-of-concept investigations. Although some platforms have evaluated clinical isolates, MIC agreement, or direct specimens, few have completed the analytical, prospective clinical, and workflow validation required for routine microbiology.

Finally, prospective multicenter studies using consecutive specimens from the intended use population are required to determine performance under routine conditions and whether the method reduces time to effective therapy without increasing false-susceptible results. Reproducibility across sites and operators, regulatory compliance, sustainable quality control, and workflow compatibility are also necessary for implementation (International Organization for Standardization, 2021; Jayakumar et al., 2022; Sounderajah et al., 2025).

### 3.3 From isolates to communities

Most FTIR and Raman based AI models have been developed using monocultures, in which each spectrum can be linked to a single bacterial species or strain. Although this approach is appropriate for controlled experiments, it may not reflect the complexity of polymicrobial infections. In a mixed sample, the resulting spectrum combines signals from organisms that may differ in abundance, physiological state, and response to treatment. Changes in community composition may therefore alter the spectral profile even when the phenotype of each organism remains unchanged. FTIR spectroscopy has successfully distinguished a defined 1:1 mixture of *Escherichia coli* and *Pseudomonas aeruginosa* from the corresponding pure cultures, but this does not establish performance in communities with unequal or changing species proportions (Al-Qadiri et al., 2006).

The problem is not limited to overlapping spectral signals. In a microbial community, antimicrobial susceptibility may depend on interactions between neighboring organisms. Resistant bacteria can protect more susceptible cells by degrading an antimicrobial, modifying the local environment, or changing community physiology. In a model of cystic fibrosis lung infection, multidrug-resistant *Stenotrophomonas maltophilia* protected susceptible *P. aeruginosa* through β-lactamase-mediated inactivation of imipenem, increasing the MIC by up to 16-fold. This effect depended on the abundance of *S. maltophilia* and was not reproduced with meropenem (Bottery et al., 2022). Thus, a model trained on monocultures may correctly identify the intrinsic phenotype of each organism but fail to predict how the community responds to treatment.

Mixed strain populations and heteroresistance create a similar difficulty. A small resistant subpopulation may be hidden within a predominantly susceptible population and contribute too little to influence a bulk or population-averaged spectrum. Single-cell studies have shown marked variation in bacterial growth and resistance gene expression during antimicrobial exposure (Wang et al., 2014).Raman-based AST has also identified borderline and hetero-resistant phenotypes in clinical isolates. Modeling from this work suggested that resistant subpopulations occurring at frequencies as low as 10–^6^ could potentially be detected, although longer incubation would be required (Bauer et al., 2020). In addition, Raman-integrated mid-infrared photothermal microscopy has visualized heterogeneous responses to antibiotics at the single-cell level (Xu et al., 2020).

Models intended for polymicrobial samples should therefore be validated using predefined mixtures that vary in species or strain composition, resistance mechanisms, and relative abundance. These mixtures should be examined both before and after antimicrobial exposure. Predictions should be compared with species-resolved reference methods, such as selective culture, population analysis profiling, molecular identification, and isolating specific MIC measurements. Studies should also report the lowest abundance at which a resistant organism or subpopulation can be detected and determine whether the model can distinguish intrinsic resistance from protection provided by neighboring cells.

Several strategies may help address these challenges, including spectral deconvolution, multilabel classification, species-specific cell separation, and single-cell analysis. Stimulated Raman scattering combined with machine learning has provided species-resolved AST of mixed *E. coli* and *Staphylococcus aureus* populations in urine within approximately 3 h Outside vibrational spectroscopy, single-cell AST followed by fluorescence *in situ* hybridization has resolved the susceptibility of individual species in mixed samples, illustrating the value of linking each phenotypic response to microbial identity (Kandavalli et al., 2022). These studies provide important proof of concept, but broader validation across clinically representative polymicrobial infections is still required.

The promise of vibrational spectroscopy and artificial intelligence lies not only in faster microbial classification but also in their ability to reveal antimicrobial resistance as a dynamic and heterogeneous biological process. Realizing this potential will require the field to move beyond accuracy measured in controlled monocultures toward biologically interpretable models, standardized workflows, diverse clinical isolates, polymicrobial samples, and prospective validation against reference AST. This effort is worthwhile because spectroscopy may bridge the gap between genetic potential and the physiological state expressed during antimicrobial exposure, allowing early adaptive responses to be recognized before stable resistance becomes established. With rigorous validation, transparent modeling, and close integration with microbiology, these technologies could evolve from promising spectral classifiers into valuable tools for understanding bacterial adaptation and supporting more timely, informed antimicrobial therapy.

## 4 Conclusion and outlook

Vibrational spectroscopy combined with chemometrics and artificial intelligence provides a promising framework for characterizing biochemical and physiological responses of bacteria to antimicrobial exposure (Kochan et al., 2019; Barrera Patiño et al., 2025; Bonner et al., 2025). FTIR, Raman-based approaches, and emerging single-cell platforms offer complementary information across different analytical scales, while computational methods can support pattern recognition, susceptibility-associated classification, temporal analysis, and hypothesis generation (Tao et al., 2017; Baker et al., 2018; Zhang et al., 2020). Nevertheless, spectral alterations associated with antimicrobial exposure should not be interpreted as direct evidence of resistance or as substitutes for clinically validated antimicrobial susceptibility testing (International Organization for Standardization, 2021; Zimmer et al., 2024; James et al., 2025).

Future progress will depend on standardized sample preparation and spectral acquisition, transparent and reproducible computational pipelines, independent external validation, and direct comparison with accepted reference AST methods and clinical breakpoints (Baker et al., 2018; Butler et al., 2019; International Organization for Standardization, 2021; Zimmer et al., 2024; James et al., 2025). Studies involving diverse clinical isolates, relevant biological matrices, polymicrobial samples, and multicenter prospective designs will be particularly important for establishing generalizability and clinical utility (Yang et al., 2019; Zhang W. et al., 2022). Single-cell spectroscopy, multimodal vibrational approaches, and explainable AI may further improve biological resolution, provided that computational attribution is supported by complementary experimental validation (Tao et al., 2017; Rudin, 2019; Zhang et al., 2020; Ghassemi et al., 2021). Collectively, these developments may advance vibrational spectroscopy from a predominantly mechanistic and proof-of-concept platform toward a more robust tool for antimicrobial-response phenotyping and, ultimately, clinically relevant microbiological investigation.
